# Supplementary material for: Causal Relationship between Aging and Anorexia Nervosa: A White-Matter-Microstructure-Mediated Mendelian Randomization Analysis
Source: Biomedicines. 2024 Aug 16;12(8):1874. doi: 10.3390/biomedicines12081874 (PMC11351342; doi:10.3390/biomedicines12081874)
Supplement: Supplementary file 1 [file biomedicines-12-01874-s001.zip › Supplementary Figure/Figure S5/Scatter plot of MR analysis for ubm-b-1840 to AN.pdf]

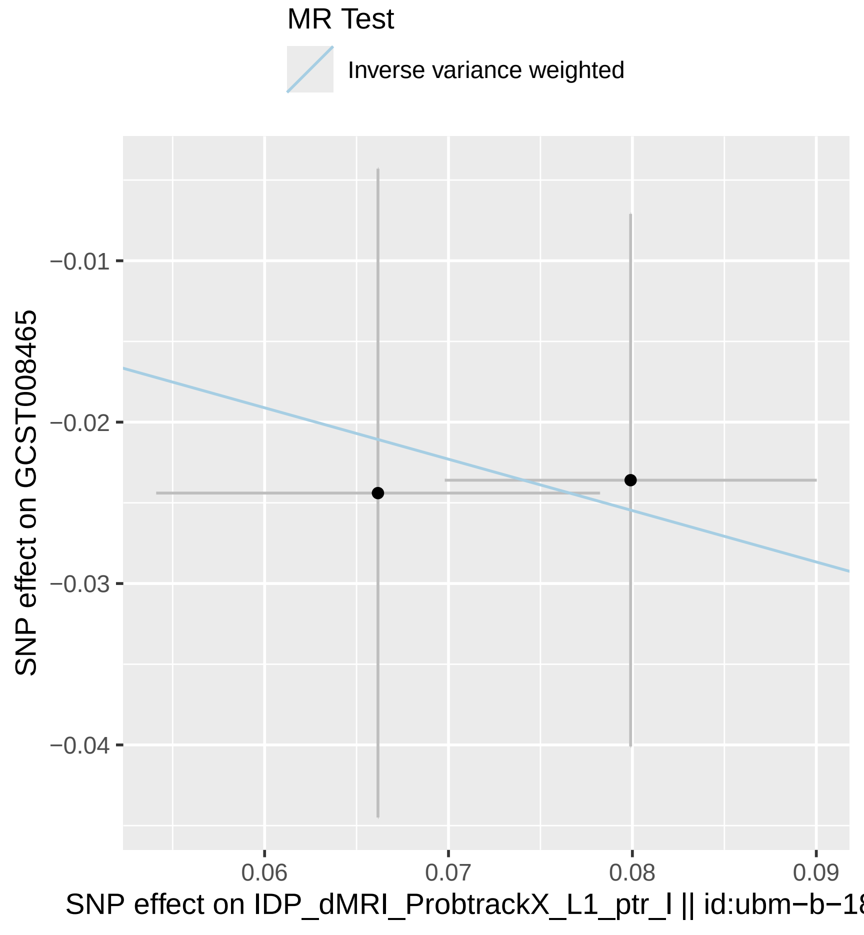
MR Test
Inverse variance weighted
−0.01
−0.02
−0.03
−0.04
SNP effect on GCST008465
0.06
0.07
0.08
0.09
SNP effect on IDP_dMRI_ProbtrackX_L1_ptr_l || id:ubm−b−18
